# Unilateral Linear Lichen Planus Pigmentosus Responding to Isotretinoin: A Case Report

**DOI:** 10.7759/cureus.33255

**Published:** 2023-01-02

**Authors:** Fadi Alghamdi, Yara Alghamdi, Abdulellah I Aleissa, Wasan AlQurashi, Abdullah Alharbi

**Affiliations:** 1 Department of Dermatology, King Fahad Armed Forces Hospital, Jeddah, SAU; 2 King Abdullah International Medical Research Center, Ministry of the National Guard - Health Affairs, Jeddah, SAU; 3 College of Medicine, King Saud Bin Abdulaziz University for Health Sciences, Jeddah, SAU; 4 College of Medicine, King Abdulaziz University Faculty of Medicine, Jeddah, SAU; 5 Department of Dermatology, King Abdullah University Hospital, Jeddah, SAU; 6 Department of Pathology, King Fahad Armed Forces Hospital, Jeddah, SAU

**Keywords:** clinical dermatology, lichen planus pigmentosus, retinoid, pathology derm, derm path

## Abstract

Lichen planus pigmentosus (LPP) is a rare variant of lichen planus. Due to the scarce number of patients diagnosed with LPP, there are no treatment guidelines. Multiple topical and oral agents are utilized in LPP with varying degrees of response. Isotretinoin has only been investigated in a case report and a single prospective pilot study for managing LPP. Herein, we report the efficacy, safety, and moderate improvement of LPP patients on isotretinoin 20 mg (0.25 mg/kg), topical adapalene gel, 4% hydroquinone cream, and topical sunscreen.

## Introduction

Lichen planus pigmentosus (LPP) is a rare variant of lichen planus (LP) that manifests in a Blaschkoid linear pattern and has been associated with genetic mosaicism mutations [1]. It manifests as dark brown-grayish macular discoloration over sun-exposed areas, along with pruritus. Treatment is quite challenging, as patients with LPP develop an inflammatory lichenoid reaction with pigment incontinence [1]. Topical treatments include medium-to-high potency corticosteroids, tacrolimus 0.03 or 0.1%, and de-pigmentation agents such as 4% hydroquinone, Konica acid, and Kligman’s formula (hydrocortisone, 4% hydroquinone, and tretinoin 0.025%-0.05%). On the other hand, systemic therapies include oral steroids, dapsone 100 mg, and isotretinoin 0.3 mg/kg [1]. Herein, we present a case of LPP that improved with a combination of oral isotretinoin 20 mg OD (0.25 mg/kg), adapalene gel, 4% hydroquinone cream, and sunscreen.

## Case presentation

**Figure 1:**
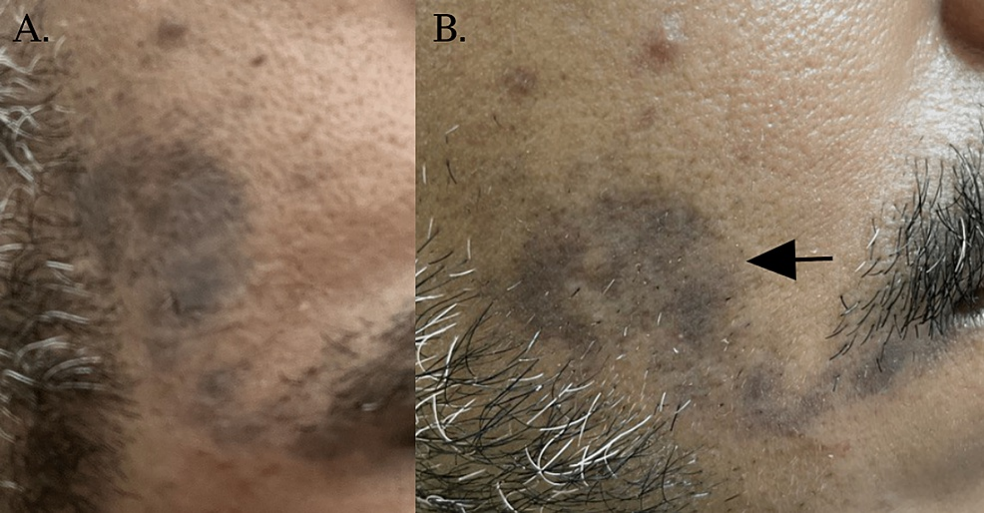
LPP in the right cheek extending to the lateral border of the lip (A) Over the right cheek, near the nasolabial fold, there are multiple, lichenoid, brown-gray macules coalescing into a patch extending to the lateral lip in a linear pattern. (B) At the three-month follow-up, over the right cheek and near the nasolabial fold, there is an improvement over the periphery showing a non-hemogenous brown-gray linear patch (arrow).

**Figure 2:**
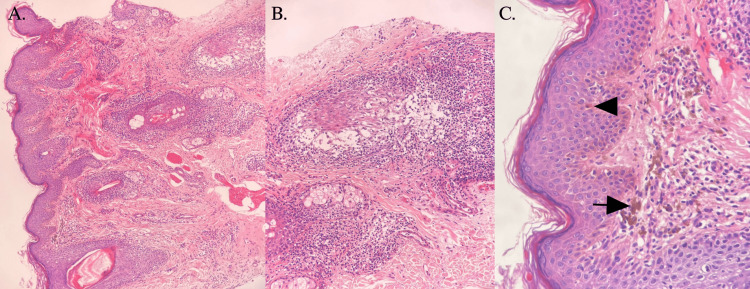
Histopathology (A) Hematoxylin and eosin (H&E) histopathology showing orthokeratosis, mild acanthosis (H&E x10). (B) Histopathology showing periadnexal inflammatory cell infiltrate (H&E x20). (C) Histopathology showing a Civatte body (arrowhead), melanin incontinence (arrow), and mild inflammatory infiltrate, suggestive of lichen planus pigmentosus (LPP) (H&E x40).

A 40-year-old male, with type-2 diabetes mellitus on metformin 1500 mg, hypertension on perindopril 10 mg, and dyslipidemia on atorvastatin 20 mg presented to the dermatology clinic with hyperpigmentation over the right cheek for four months. The dermatological assessment showed multiple, coalescing, brown-to-grayish macules and papules forming a patch over the right cheek (Figure 1A); there was no erythema or scales. Histopathology showed acanthosis and mild hyperkeratosis with melanophages along with peri- and intrafollicular inflammatory cell infiltrate in the dermis (Figure 2). The clinical and histopathological evaluation confirmed LLP. Labs for liver function test, lipid profile, and blood chemistries were all satisfactory to start oral isotretinoin 20 mg OD (0.25 mg/kg). Topical treatments were adapalene gel and 4% hydroquinone cream. Sun protection along with re-application was instructed to the patient. At the three-month follow-up examination, the hyperpigmentation improved (Figure 1B), and the patient will continue oral isotretinoin 20 mg OD along with topical adapalene gel and 4% hydroquinone cream.

## Discussion

LPP is a difficult entity to manage. To date, there is no treatment algorithm to guide management. Only case reports and case series have reported that administered therapy can help achieve improvement. Isotretinoin is a 13-cis-retinoic acid that over-expresses apoptotic protein and necrotic factors and increases cell turnover [2]. It is administered in a variety of conditions such as acne, hidradenitis suppurativa, pustular psoriasis, chronic plaque psoriasis, papulopustular rosacea, seborrheic dermatitis, folliculitis decalvans, lichen planus, and photo-aging [1,2]. There were few case reports that investigated the efficacy of isotretinoin in LPP [3-6].

LPP has been described as a variant of LP after the investigation of a subset of Indian patients with dyschromicum perstans (EDP) (ashy dermatosis), which yielded clinical and histopathological evidence of lichen planus [3]. The first report that utilized vitamin A in LPP was by Bhutani et al. back in 1979 who reported 88.5% of Indian patients have shown varying degrees of improvement on vitamin A [3]. Shah et al. also reported a case of a 46-year-old female with LPP who was stabilized at two months after isotretinoin initiation and achieved near-complete resolution at nine months [4]. On the other hand, Wolff et al. reported an improvement of LPP over the face, neck, and both hands in an 18-year-old male utilizing 5% azelaic acid foam, tretinoin 0.1%, and chemical peels. A prospective pilot study investigating the efficacy and safety of low-dose isotretinoin in LPP, conducted at a pigmentary clinic in Chandigarh, India, reported that 55.7% of the studied population achieved moderate improvement while 21.8% and 6.2% had achieved good and mild outcomes, respectively [6].

The patient in this report had achieved a moderate improvement within three months, which is considered an accelerated response as compared with other published cases. We believe the reason behind the expedited response is a combination of topical adapalene gel and isotretinoin 20 mg (0.25 mg/kg). In addition, the low-dose isotretinoin was tolerable in this patient with hyperlipidemia, as triglyceride levels remained within the normal range. There were no complaints of xerosis. In the literature, there are no reports of LPP that was managed with a combination of topical and oral vitamin A derivatives along with a depigmentation agent, 4% hydroquinone cream.

## Conclusions

LPP is a difficult entity to manage. A combination of topical adapalene gel and low-dose isotretinoin 20 mg (0.25 mg/kg), topical sunscreen, and 4% hydroquinone cream showed promising and accelerated results within three months. Usually, a similar response can be achieved at six months. Thus, a single agent would not achieve a desirable outcome, and a combination of topical and oral therapies is the key for managing a perplexing disease, LPP.
